# Comparative and integrative analysis of transcriptomic and epigenomic-wide DNA methylation changes in African American prostate cancer

**DOI:** 10.1080/15592294.2023.2180585

**Published:** 2023-02-22

**Authors:** Chad J. Creighton, Flora Zhang, Yiqun Zhang, Patricia Castro, Rong Hu, Md Islam, Somiranjan Ghosh, Michael Ittmann, Bernard Kwabi-Addo

**Affiliations:** a Dan L. Duncan Comprehensive Cancer Center, Baylor College of Medicine, Houston, TX, USA; b Center for Women’s Studies, Colgate University, Hamilton, New York, USA; c Department of Pathology and Immunology, Michael E. DeBakey Veterans Affairs Medical Center, Houston, Texas, USA; d Lombardi Comprehensive Cancer Center, Georgetown University, Washington, District of Columbia, USA; e Department of Biology, Howard University, Washington, Columbia, USA; f Department of Biochemistry and Molecular Biology, Howard University, Washington, Columbia, USA

**Keywords:** Clinically localized prostate cancer, DNA methylation, gene expression, Genome-wide profiling, CpG methylation, African American men

## Abstract

African American (AA) men have the highest incidence and mortality rate from Prostate cancer (PCa) than any other racial/ethnic group. To date, PCa genomic studies have largely under-represented tumour samples from AA men. We measured genome-wide DNA methylation in benign and tumor prostate tissues from AA men using the Illumina Infunium 850 K EPIC array. mRNA expression database from a subset of the AA biospecimen were used to assess correlation of transcriptome and methylation datasets. Genome-wide methylation analysis identified 11,460 probes that were significant (p < 0.01) and differentially methylated in AA PCa compared to normal prostate tissues and showed significant (p < 0.01) inverse-correlation with mRNA expression. Ingenuity pathway analysis and Gene Ontology analysis in our AA dataset compared with TCGA dataset showed similarities in methylation patterns: top candidate genes with significant hypermethylation and corresponding down-regulated gene expression were associated with biological pathways in hemidesmosome assembly, mammary gland development, epidermis development, hormone biosynthesis, and cell communication. In addition, top candidate genes with significant hypomethylation and corresponding up-regulated gene expression were associated with biological pathways in macrophage differentiation, cAMP-dependent protein kinase activity, protein destabilization, transcription co-repression, and fatty acid biosynthesis. In contrast, differences in genome-wide methylation in our AA dataset compared with TCGA dataset were enriched for genes in steroid signalling, immune signalling, chromatin structure remodelling and RNA processing. Overall, differential methylation of *AMIGO*3, *IER*3, *UPB*1, *GRM*7, *TFAP*2C, *TOX*2, *PLSCR*2, *ZNF*292, *ESR*2, *MIXL*1, *BOLL,* and *FGF*6 were significant and uniquely associated with PCa progression in our AA cohort.

## Introduction

Prostate cancer is the second most commonly diagnosed cancer in men after skin cancer. The American Cancer Society estimates in 2021 that 248,530 men will be diagnosed with prostate cancer (PCa) and 34,130 men will die of their disease[1]. Population-based estimates demonstrate that African American (AA) men are more likely to be diagnosed with PCa, more likely to present with distant metastases, and nearly 2.5 times more likely die of the disease when compared with European American (EUR) men[1]. African American men with PCa have lower socioeconomic status[2] are diagnosed at a later stage[3] and receive less guideline-concordant care[4]. Differences in outcomes persist even after correcting for socioeconomic covariates [3,5]. At the molecular level, PCa is a complex and heterogenous disease, characterized by complex genetic and epigenetic alterations and documented differences in molecular profiles in different racial/ethnic groups may contribute to the disparities associated with disease aetiology and/or progression. Differential somatic alterations in tumours across different race and ethnicities has been shown to have clinical implications. Several studies have explored the molecular basis of primary PCa and have identified multiple recurrent genomic alterations that include mutations, DNA copy-number changes, rearrangements, and gene fusions [6–8] with varying degrees of DNA copy-number alteration; indolent and low-Gleason tumours have few alterations, whereas more aggressive primary and metastatic tumours have extensive burdens of copy-number alteration genome-wide [9,10]. In contrast, somatic point mutations are less common in PCa than in most other solid tumours. The most frequently mutated genes in primary PCa are *SPOP, TP53, FOXA1*, and *PTEN*[11]. Overall, the most common alterations in PCa genomes are fusions of androgen-regulated promoters with *ERG* and other members of the ETS family of transcription factors. In particular, the *TMPRSS2-ERG* fusion is the most common molecular alteration in PCa; found in 40 to 50% of all prostate tumour foci[12]. Yet, there is significant molecular differences between PCa in AA and EA. The *TMPRSS2-ERG* fusion and deletion in *PTEN* were less frequent in tumours from AA men, whereas *MYC* amplifications were more frequent[13]. The Spink1 overexpression, an alteration associated with more aggressive PCa was more frequent in AA, while *ERG* rearrangement and *PTEN* deletion were less frequent in this cohort[14]. Notably, *FOXA1* mutations are highly prevalent among Asians with prostate cancer while being less frequent in prostate tumours from EUR men[15]. There is paucity of data about the genetic alterations associated with prostate cancer risk in Hispanic/Latino men. However, a study by Bree et al. has identified genetic alterations in *BRCA*2 and *MSH*6 to be associated with increased risk of prostate cancer in Hispanic/Latino men[16]. Further investigation is needed to determine whether these molecular differences explain some of the disparity in incidence and mortality between different ethnic groups.

Only recently has the spectrum of epigenetic changes in PCa genomes been explored [17,18]. DNA methylation of CpG sites (CpGs) is a well-known epigenetic mechanism for controlling gene expression[19]. Epigenetic patterns are known to be altered in several different cancer types, including PCa, and signatures of DNA methylation may serve as potential diagnostic or prognostic biomarkers[20]. Previous studies investigating DNA methylation patterns at select genomic loci in PCa resulted in discoveries of epigenetic differences between PCa tissue and normal-adjacent prostate in genes such as glutathione s-transferase 1 (*GSTP1*), Ras association domain family member 1 (*RASSF1*), and adenomatous polyposis coli (*APC*), among others [21–23]. Cancer-derived, methylated DNA has been identified and purified from both patient serum and urine, making it a promising option for a non-invasive biomarker[24]. A review of several studies for the role for DNA methylation in PCa development[25], research has identified several methylation markers to be potentially associated with PCa risk, such as methylation at *GSTP1, CDKN2A, DNMT3B, SCGB3A1*, and *HIF3A* [25–29]. This earlier work led to the development of an epigenetic test that measures the methylation levels of three genes, *GSTP*1, *APC*, and *RASSF*1, for the detection of PCa[30]. The epigenetic assay, in combination with other known risk factors, may help reduce unnecessary repeat prostate biopsies and identify men at highest risk of harbouring occult high-grade prostate cancer[31]. Few studies have examined differences in aberrant DNA methylation in AA and EUR PCa patients. Some of the early studies were focused on candidate gene studies [32–38]. For instance, we have reported higher frequency of hypermethylation of *GSTP*1, *NKX*2-5, and *TIMP*3 in AA PCa compared to EUR PCa[36], and Woodson et al.[32] described differential hypermethylation of *CD*44 in AA PCa versus EUR PCa. Similarly, Tang et al.[37] reported that hypermethylation of *RAR*β2 was significantly associated with a higher risk of PCa in AA men but not in EUR men. Several recent studies have taken a more comprehensive approach to investigate DNA methylation alterations in PCa using array-based platforms with varying degrees of epigenome-wide coverage such as the Illumina Infunium Human Methylation 27 (HM27) and HumanMethylation450 (HM450) BeadChip, which integrates regions outside gene promoters such as gene body and intergenic regions. Using the HM450 BeadChip array, we found the promoter methylation of several genes; including *SNRPN, MST*1R, and *ABCG*5, were differentially hypermethylated in AA PCa compared to EUR PCa[39] suggesting that the differential methylation levels may influence gene expression and disease milieu in AA compared to EUR PCa. Another study reported epigenome-wide DNA methylation analysis of 76 AA PCa patients and identified methylation differences between metastatic-lethal PCa versus no recurrence, regional versus local pathological stage, and higher versus lower tumour aggressiveness indicating that differential methylation identified in tumour tissues of AA men may contribute to PCa aggressiveness.

Given the considerable and persistent racial disparities in prostate cancer outcomes, better understanding of the epigenetic mechanisms underlying these disparities are needed to provide more insights into the molecular mechanisms of the disease, identify novel DNA-based methylation biomarkers and develop new strategies to overcome PCa disparity. Importantly, there are very few studies that have comprehensively analysed the transcriptome and epigenome of AA PCa. The primary goal of the present study is to carry out Epigenome-wide DNA methylation changes in 113 prostate tissues from AA to identify biological pathways and genetic alterations associated with AA PCa, compare differential methylation changes with AA mRNA expression dataset to see whether differences corresponded with changes in mRNA expression and to comprehensively interrogate our AA methylation and mRNA expression (Howard/BCM) dataset with the TCGA (EUR and AA) methylation and mRNA expression dataset for differences in the two datasets.

## Methods

*Human Subjects* – This human study was approved by the Institutional Review Board (IRB) of the Baylor College of Medicine (H-14435; Houston TX) and by the Howard University (IRB-19-MED-08; Washington DC). Tissue samples were obtained from the Human Tissue Acquisition and Pathology Core of the Dan L. Duncan Cancer Center and were collected from fresh radical prostatectomy specimens after obtaining informed consent under an Institutional Review Board approved protocol. The study enrolled 113 self-identified African American men who had undergone radical prostatectomy and genome DNA were extracted from freshly frozen tissues as previously described[40]. DNAs were extracted using Qiagen DNA Mini kit according to the manufacturer’s instruction (Qiagen Inc. GermanTown, MD). Cancer tissues contained at least 70% cancer and benign tissues were free of cancer or high-grade prostatic intra-epithelial neoplasia as confirmed by frozen section before DNA extraction. All tissues were from self-identified AA men. Table 1 highlights the clinicopathological variables of the enrolled patients, age and Gleason grade group. The mean age of the 113 patients in the study was 57.7 years old.

**Table 1.** Clinicopathological data of the 113 AA prostate tissues. Number and percentage (in parentheses) of all cancer cases are shown. The age range and mean age, median, standard error (Std. Err), standard deviation (Std. dev) as well as the 95% confidence interval for mean are shown. Independent samples t-test shows that means of age are not significantly different for Grade groups 1, 2 and 3. Independent samples median test reveals that the medians of age are not significantly different for Gleason grade 4 and 5 (Fisher Exact test, case sizes are too small for Group 4 and 5 to make comparison with Group 1–3).

	Number (Percent)	Age Range (years)
Gleason score		
5 and 6	16 (25)	45–70 (57.62 + 6.04)
7–10	47 (75)	45–73 (57/78 + 7.08)

*Genome-wide DNA methylation analysis*- DNA (0.5 µg) was bisulphite converted using the EZ DNA Methylation Gold kit (Zymo Research, Irvine, CA). The converted DNA was then whole genome amplified, enzymatically fragmented, precipitated using Infinium HD Assay Methylation protocol (Illumina, San Diego, CA). The re-suspended samples were hybridized at 48°C for 20 h to Infinium Methylation Epic BeadChip which contains over 850,000 methylation sites per sample at single-nucleotide resolution. After hybridization, the Cytosine or Thymine nucleotides were detected by fluorescent single-base primer extension using Illumina iScan or NextSeq550. Initial array results were visualized using Illumina® GenomeStudio Methylation Module (Version 2011.1). The methylation score for each CpG site is represented as a β value. The β value is a continuous variable ranging between 0 and 1, representing the ratio of the intensity of the methylated-probe signal to the total locus signal intensity. A β value of 0 corresponds to no methylation while a value of 1 corresponds to 100% methylation at the specific CpG site measured. These values were used to calculate a ratio of relative methylation between samples, with higher values corresponding to greater levels of methylation in tumour tissue relative to normal. CpG methylation differences examined were above a cut-off P value < 10 ^−^[3] and 0.2-fold change in the β value, unless specifically indicated otherwise. All other computations and statistical analyses were performed using Partek Genome Studio. For downstream pathway analysis, we exported the candidate gene list from Partek to IPA.

*Gene expression microarray analysis*- The African American gene expression analysis is previously described[41]. The expression array analysis was performed using RNAs from 44 high purity tumours from AA men and 47 normal prostate tissues using Agilent 60 K expression arrays. Briefly, twenty-five ng of total RNA was amplified and labelled with Cy3 dye using Low Input Quick Amp Labelling Kit (Agilent Technologies). For microarray hybridization, 825 ng of cyanine 3-labelled cRNA was fragmented and hybridized on the Agilent SurePrint G3 Human GE 8 × 60 K V2 Microarrays at 65°C for 17 hours using the Agilent Gene Expression Hybridization Kit. The processed microarrays were scanned Agilent High-Resolution SureScan microarray scanner and data was extracted using Agilent Feature Extraction Software (11.5.1.1). This expression array is hereafter referred to as the Baylor AA mRNA dataset.

*Data Analysis*- For DNA methylation data, differences between comparison groups were evaluated using t-tests on logit-transformed data. For mRNA data, differences between comparison groups were evaluated using t-tests on log2-transformed data. Enrichment of GO annotation terms within sets of differentially expressed genes was evaluated using SigTerms software[42] and one-sided Fisher’s exact tests. Expression patterns were visualized as colour maps using Java TreeView[43].

## Results

**Figure 1.**
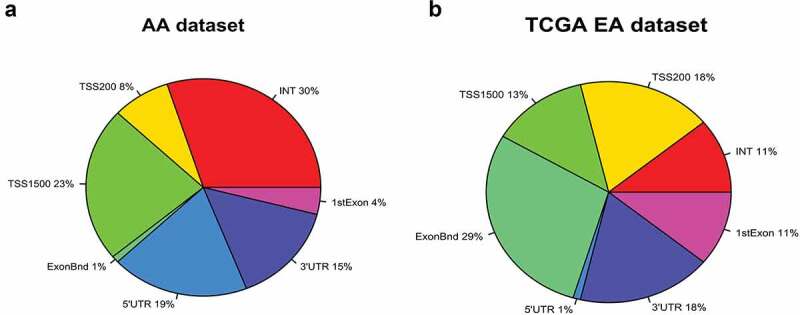
Genomic distribution of 5623 top differentially methylated CpG probes in prostate cancer compared to normal prostate tissues is expressed as percentage landscape distribution in a pie chart. **a**. Analysis in HUBCM dataset. **b**. Analysis in TCGA dataset. The methylation levels of CpG probes are shown by functional locations including TSS1500, TSS200, 5ʹUTR, 1st Exon, Body, and 3ʹUTR and intergenic (INT) regions.

*Comparative analysis of differential genome-wide DNA methylation and transcriptome analysis in Howard-BCM (HUBCM) dataset and TCGA dataset*. We have carried out genome-wide DNA methylation on 113 prostate tissues (63 tumours and 50 normal prostate tissues) from AA patients and compared the differential methylation level of PCa versus normal tissues with the Baylor (BCM) AA mRNA (44 tumours and 47 normal prostate tissue samples) dataset. This initial analysis revealed 11,460 significant (p < 0.01) differential methylation probes in AA PCa compared to AA normal tissues and showed significant (p < 0.01) and inverse correlation with the AA mRNA expression data[41] (data not shown). It is possible that some of the candidate DNA methylation probes may be uniquely methylated in ethnic-specific manner as we and others have reported that DNA methylation in prostate vary across ethnicities [32,36,37,39]. Thus at least some PCa associated CpG sites identified using the Illumina genome-wide array should be ethnic-specific. Therefore, we compared our HUBCM dataset with the TCGA dataset[44]. Although, our dataset was generated from Illumina EPIC array, whereas the TCGA dataset is from the 450 K Illumina, by joining these two different array platforms using the Illumina CpG loci identifier, we were able to accurately analyse the same CpG loci across the two studies. Comparative analysis of the top 11,460 probes in the HUBCM dataset with the TCGA dataset revealed 5623 top differentially methylated CpG probes (P < 0.01) in PCa versus normal tissues that were inversely correlated with RNA expression (p < 0.01) in both the HUBCM and TCGA datasets. The genomic landscape indicates that about 20% of CpG sites are located in CpG-rich (CpG islands) areas while the rest mapped to regions of lower CpG density. We therefore mapped the genomic distribution of the 5623 top differentially methylated CpGs and is expressed as percentage genomic distribution of CpG probes in a pie chart landscape of our AA dataset compared to the TCGA dataset (Figure 1). Among the top differentially methylated CpGs in the HUBCM dataset, 54% were localized in the promoter region that corresponds to regions TSS1500, TSS200, and 5ʹUTR and 1st Exon whereas greater number of the differentially methylated CpG in the TCGA EUR dataset (71%) were located in the promoter region. In addition, the HUBCM differentially methylated CpGs appears to have 30% in the noncoding region (Intergenic, INT) followed by 15% in the 3ʹUTR whereas the TCGA only has 18% in the intergenic and 11% in the 3ʹUTR.

In Table 2, Gene Ontology (GO) term search for functional categories and cellular localization of genes associated with the top inverse differential methylation and expression in both the HUBCM and the TCGA AA datasets and yielded two sets of results for genes enriched in various biological processes: one for the gene with high methylation and low expression (Table 2) and the other set for the genes with low methylation and high expression (Table 2). The top five significant canonical pathways enriched in this dataset presented in the GO term that are differentially hypermethylated with corresponding decrease in mRNA expression are genes that function in biological pathways in hemidesmosome assembly, mammary gland development, epidermis development, hormone biosynthetic process and cell communication. Top candidate genes identified in these pathways are *COL*7A1, *ITGB*4, *KRT*5, *LAMA*3, *LAMB*3, *B4GALT*1, *NRG*1, *IRS*1, *NOTCH*4, and *TGFB*3. Similarly, the top five significant canonical pathway in the GO term that are differentially hypomethylated with corresponding increase in mRNA expression are genes that function as negative regulators of macrophage differentiation, cAMP-dependent protein kinase inhibitor activity, protein destabilization, clathrin-coated vesicle membrane as well as transcription corepressor activity. Top candidate genes in these pathways are *INHBA, C1QC, PRKAG*2, *PKIB, MYLIP, PRKDC, DAB*2, *DENND*1A, *TFEC* and *HOXC*6.

**Table 2.** GO term search results showing the functional categories for the genes associated with differential methylation and expression. **A**. Genes with high methylation and low expression and **B**. Genes with low methylation and high expression. For each result set, there is GO functional category, name of enrichment term, number of genes counted in term, then the number of counts in total population and the biological genes involved as well as the degree of statistical enrichment.

Functional Category	Name of Enrichment Term	Count of selected Genes	Count in Total Population	Involved gene list	p-value
Table 2A					
GO	hemidesmosone assembly	6	11	COL17A1, ITGB4, KRT5, KRT4, LAMA3, LAMB3	5.29E-06
GO	mammary gland development	8	26	B4GALT1, NRG1, IRS1, NOTCH4, TGFB3, CAV1, WNT3A, NTN1	2.4E-05
GO	epidermis development	14	81	COL7A1, COL17A1, ALDH3A2, GJB5, KRT5, KRT13, KRT14, KRT15, KRT17, LAMA3, LAMB3, ATP2A2, NTF3, GRHL3	3.8E-05
GO	hormone biosynthetic process	5	10	HFE, DUOX2, DUOX1, TG, CHST9	5.91E-05
GO	cell communication	14	89	NOXO1, GJB4, SNX31, GJB3, GJB5, NRG1, ITGB4, JAG2, SLC8A2, SNX1, KREMEN1, PPFIBP2, SNX21, TRIP10	0.00011
GO	response to hypoxia	18	140	CHRNA7, EPAS1, PTK2B, ALDOC, HSD11B2, JAG2, SMAD3, ANGPLT4, PSEN2, PYGM, BCL2, RYR1, SCNN1B, TGFB3, VEGFA, ACTN4, CAV1, PDLIM1	0.000183
GO	positive regulation of stress fibre assembly	5	13	SORBS3, AMOT, SMAD3, NF2, SDC4	0.000267
GO	basal plasma membrane	6	21	MUC20, ITGB4, MET, DST, TF, CAV1	0.000406
GO	Wnt receptor signalling pathway, calcium modulating pathway	6	21	ROR2, WNT4, WNT6, WNT10A, WNT5B, WNT3A	0.000406
GO	endothelial cell morphogenesis	3	4	HOXA13, ID1, NOTCH4	0.000461
GO	pyruvate dehydrogenase (acetyl-transferring) kinase activity	3	4	PDK2, PDK3, PDK4	0.000461
GO	cytoskeletal protein binding	8	39	ALDOA, ALDOC, B4GALT1, ANXA2, NF2, SDC4, FRMD5, DLG5	0.000534
GO	apical plasma membrane	18	153	PDPN, SLC46A1, PROM2, CTSB, SLC5A8, MUC20, NRG1, ABCC6, SLC22A18, P2RY2, DUOX2, ATP6VOA4, DUOX1, VANGL2, SCNN1A, SCNN1B, TF, CAV1	0.000551
GO	aldehyde dehydrogenase (NAD) activity	4	9	ALDH2, ALDH3A1, ALDH3A2, ALDH4A1	0.000607
GO	focal adhesion	12	84	ARPC2, SORBS3, PTK2B, FHL2, TES, SDC4, TNS1, TRIP6, TNS4, JUB, CAV1, BCAR1	0.000823
GO	structural constituent of cytoskeleton	11	73	ARPC2, SORBS3, ADD3, DES, SYNM, KRT5, KRT14, KRT15, KRT17, KRT19, VILL	0.000853
GO	lung alveolus development	6	24	PDPN, PSEN2, SFTPD, SOX2, TGFB3, VEGFA	0.000887
GO	positive regulation of cell migration	10	63	PDPN, AKT2, PTK2B, IRS1, SMAD3, ROR2, TRIP6, VEGFA, WNT5B, BCAR1 PDPN	0.000961
Table 2B					
GO	negative regulation of macrophage differentiation	2	4	INHBA, C1QC	0.000314
GO	cAMP-dependent protein kinase inhibitor activity	2	5	PRKAG2, PKIB	0.000521
GO	protein destabilization	2	8	MYLIP, PRKDC	0.001439
GO	clathrin coated vesicle membrane	2	11	DAB2, DENND1A	0.002787
GO	transcription corepressor activity	5	140	TFEC, HOXC6, APEX1, YY1, NRIP1	0.003649
GO	fatty acid biosynthetic process	3	46	PTGES3, TMEM195, PRKAG2	0.004591
GO	phosphatidylinositol-3,4,5-trisphosphate binding	2	16	ADAP2, BTK	0.005937
GO	positive regulation of neutrophil differentiation	1	1	IK2F1	0.007302
GO	tRNA export from nucleus	1	1	XPOT	0.007302
GO	2-oxoglutarate-dependent dioxygenase activity	1	1	ALKBH2	0.007302
GO	oxidative DNA demethylation	1	1	ALKBH2	0.007302
GO	collagen type VIII	1	1	COL8A1	0.007302
GO	dUTP diphosphatase activity	1	1	DUT	0.007302
GO	dUTP metabolic process	1	1	DUT	0.007302
GO	alpha-methylacyl-CoA racemase activity	1	1	AMACR	0.007302
GO	phosphoribosylamine-glycine ligase activity	1	1	GART	0.007302
GO	phosphoribosylformylglycinamidine cyclo-ligase activity	1	1	GART	0.007302
GO	phosphoribosylglycinamide formyltransferase activity	1	1	GART	0.007302

**Figure 2.**
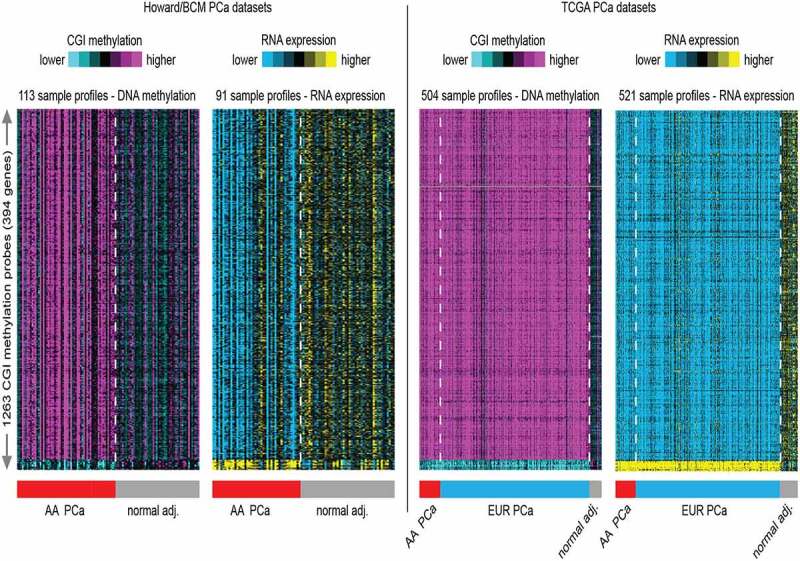
Heat map generated from hierarchical clustering analysis of the top 1263 CGI probes most variable CpG islands across all samples in the genome-wide methylation analysis and correlated with RNA expression profiles. **A**. The AA dataset consists of 63 PCa and 50 normal adjacent (adj.) prostate tissue DNA used in the methylation analysis and 96 RNA samples (matched PCa and normal adj. tissues) used in the transcription analysis. B. The TCGA methylation dataset consists of 504 prostate tissues used in the genome-wide DNA methylation analysis. This is made up of 58 AA PCa and 3 AA normal prostate tissues, and 412 EUR PCa and 31 EUR normal prostate tissues. The TCGA RNA expression consists of 521 prostate tissue RNA samples. This is made up of from 58 AA PCa and 7 AA normal prostate tissues, and 412 EUR PCa and 44 EUR normal tissues.

Next, hierarchical analysis of significant (p < 0.01) differential methylated changes greater than five percent in CpG island within a 2 kb promoter region important for regulating the expression of genes[45] in the HUBCM PCa compared to normal tissues identified 1263 CpG islands (CGI) probes representing 394 genes for which there are significant (p < 0.01) and corresponding inverse correlation with gene expression (Figure 2; Howard/BCM datasets). An unbiased analysis of these probes was examined in the TCGA dataset. The TCGA methylation dataset was derived from 58 AA PCa and 3 AA normal prostate tissues, and 412 EUR PCa and 31 EUR normal prostate tissues. The TCGA RNA expression dataset was derived from 58 AA PCa and 7 AA normal prostate tissues, and 412 EUR PCa and 44 EUR normal tissues. Overall, the TCGA datasets trends broadly agreed well with results from the HUBCM dataset results, whereby we identified hypermethylated probes, this corresponded with downregulated genes and similarly hypomethylated genes corresponded with upregulated genes after overlapping the HUBCM and the TCGA datasets. The top 10 significant genes that demonstrated differential CGI hypermethylation and corresponding decrease mRNA in PCa compared to normal tissues are *CDH*3, *HDAC*5, *ARPC*2, *MBNL*2, *WASF*2, *RASGRP*2, *CDKN*1C, *MRVI*1, *PGCP*, and *YAP*1. Similarly, top candidate genes with differential CGI hypomethylation and corresponding increased mRNA expression in PCa compared to benign tissues are *XPOT, MARS, METTL*1, *XPO*5, *EIF*2A, *MTHFD*2, *MTHFD*1L, *MTHFD*2L, *EIF*5A, and *SEC61A*1.

**Figure 3.**
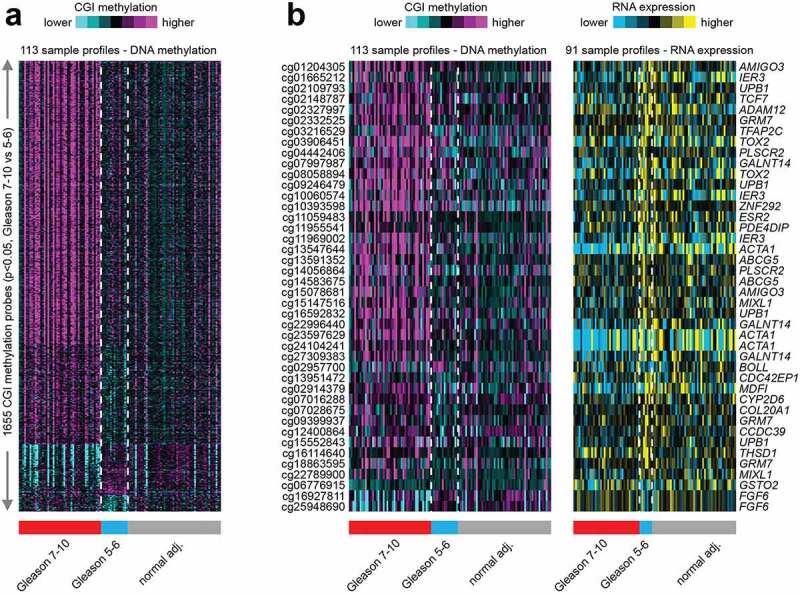
Hierarchical clustering analysis of 1655 CGI probes across all AA prostate tissue samples categorized by Gleason score 7–10 (grade 2–5) tumours, Gleason score 5–6 (grade 1) tumours and normal (benign) prostate tissues. **a**. Clustering analysis of the top 1655 CGI differential methylation that are significant (p < 0.05) by t-test and methylation change greater than 5%) and separated into Gleason 7–10; Gleason 5–6; and benign prostate tissues. **b**. Comparison of the significant differential DNA methylation and mRNA expression as categorized by Gleason score and normal tissues.

**Figure 4.**
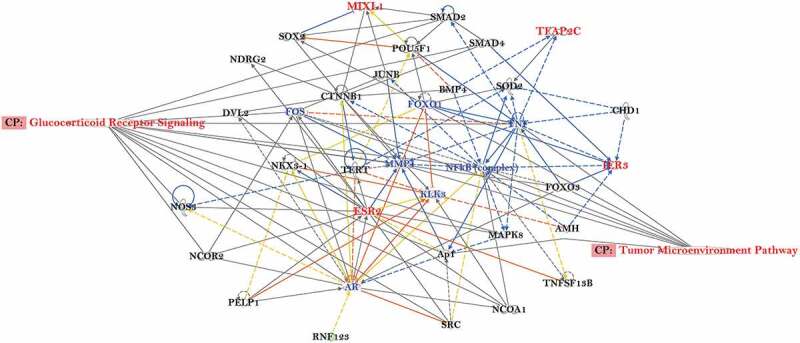
Predicted gene regulatory network of the overlapping genes associated with increasing Gleason score in AA prostate cancer. Ingenuity Pathway Analysis software was used to predict putative sub-networks containing five candidate genes: *IER*3, *TFAP*2C, *ESR*2, *MIXL*1, *AMIGO3* (*RNF*123) that play a role in several mechanisms associated steroid signalling and tumour microenvironment in prostate cancer disease progression. MIXLI, TFAP2C, IER3 and ESR2 are highlighted in red.

To investigate if DNA methylation patterns can distinguish tumour grades compared to normal tissues in the HUBCM cohort, we compared the DNA methylation pattern of high grade versus low grade prostate tumours and normal tissues (Figure 3a). Out of 161,441 CpG probes represented on the DNA methylation array platform, 1655 probes correlated significantly with Gleason score, comparing Gleason 7–10 (tumour grade 2–5) tumours with Gleason 5–6 (tumour grade 1) tumours and normal tissues (p < 0.05 by t-test and methylation change greater than 5%). We focused on the CGI probes since this is easier to associate genes and classical epigenetic silencing with these probes[46]. A subset of the 1655 CGI probes involved an associated genes with an inverse correlation to Gleason grade at the mRNA level, involving 42 probes that corresponded to 25 unique genes (Figure 3b). Overall, the genome-wide differentially DNA methylated 25 genes in the HUBCM dataset compared to TCGA dataset were similar suggesting an overall concordance between our AA cohort and the TCGA cohort. However, differential methylation of 12 genes (Table 3; *AMIGO3, IER*3, *UPB*1, *GRM*7, *TFAP*2C, *TOX*2, *PLSCR*2, *ZNF*292, *ESR*2, *MIXL*1, *BOLL, FGF*6) were significantly (P < 0.05) correlated with Gleason score in the HUBCM cohort compared to TCGA (EUR and AA) cohort. Of these genes, *BOLL* and *FGF*6 demonstrated inverse correlation between grade 2–5 versus grade 1 whereas the other 10 genes demonstrated direct correlation between grade 2–5 versus grade 1 (*AMIGO3, IER*3, *UPB*1, *GRM*7, *TFAP*2C, *TOX*2, *PLSCR*2, *ZNF*292, *ESR*2, *MIXL*1). To identify common biologic processes that potentially explain the association between AA men and prostate cancer progression, pathway analyses were performed using Ingenuity Pathway Analysis (IPA) software (Figure 4) on this subset of the genes whose differential methylation and expression levels were significantly correlated with Gleason score in the AA PCa. The top network of functional genes directly observed in the IPA pathway are *ESR*2, *IER*3, *TFAP2C*, and *MIXL*I. Other genes played indirect functional roles such as transcriptional regulators of hormonal signals (ZNF292, TFAP2C) or genes which belongs to immunoglobulin superfamily (*AMIGO*3). These genes were found to be involved in the Glucocorticoid Receptor Signalling, and Tumour Micro environment Pathway.

**Table 3.** Statistical analysis of differential methylated probes and associated gene symbol for PCa in the Howard/BCM (HUBCM) datasets and the TCGA (EUR and AA) datasets. Highlight in pink show direct correlation of differential methylation in high versus low Gleason grade PCa. Highlights in green shows inverse correlation of differential methylation in high versus low grade PCa. P-values for all probes are shown.

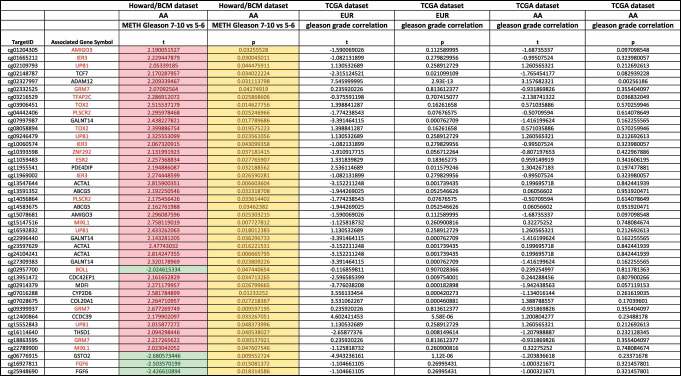

## Discussion

Previous studies have shown biological differences of PCa by race and ethnicity[47] suggesting that AA PCa harbours more Androgen Receptor alterations, more aneuploidy, particularly chromosome arm 8q gains and fewer PTEN and p53 mutations than EUR men.

In addition to the genetic alterations, there is accumulating evidence to suggest epigenetic alterations are significantly different in PCa of AA and EUR men. DNA methylation is a molecular epigenetic event that we and others have shown to differ in PCa among AA and EUR [36,39,48,49]. These studies were limited and focused on a few genes, notably tumour suppressors (*GSTP*1, *RARβ*2, *RASSF*1A, *CD*44, *CAV*1, *CDH*13, *CDH*1, and *TIMP*3), whereby differential DNA methylated mediated gene silencing of tumour suppressor genes and could contribute to aggressive disease milieu observed in AA men. Recently, the role of miRNA in racial disparities associated with PCa has been reported. Work carried out by Wang et al.[50] identified 22 miRNA signatures in AA PCa and 18 miRNAs in EUR prostate cancer whereby the miRNA-mRNA pairs predict enhanced activation of EGFR-PI3K-AKT signals in AA compared to EUR cancer.

Yet, most cancer genomic studies have largely underrepresented racial/ethnic minority groups despite the greater burden of PCa in AA men[51]. Large sample sizes from AA men are needed to detect significant association of genetic alterations and clinical outcomes in AA population. Given the limited data of AA DNA methylated data, the goal of this study was to investigate epigenome-wide DNA methylation in 63 PCa and 50 normal prostate tissue samples from AA patients. We carried out integrative analysis of our epigenetic DNA methylation changes with an existing mRNA expression data derived from AA patients (HUBCM dataset). We observed significant and varied differential methylation in AA PCa versus normal. A total of 11460 CpG probes showed significant and inverse correlation between methylation and mRNA expression. Although our RNA dataset was generated using Agilent microarray, whereas the TCGA RNA dataset was derived from multiple platforms, studies that compared different microarray technologies reported lowest generalized variance among replicates and these were highly correlated as well as showed remarkable degree of overlap in genes found to be differentially expressed, particularly for the Agilent and Affymetrix platforms [52,53]. This gave us the confidence that we could successfully compare the transcriptional profiles from our HUBCM dataset with the TCGA dataset. Comparative analysis of the HUBCM dataset with the TCGA dataset revealed that of the 11,460 CpG probes, a total of 5623 CpG probes after correcting for multiple testing were similar in the two datasets as these were significant and differentially methylated in cancer versus normal tissues and showed inverse correlation with RNA expression. Gene ontology analysis showed highly significant enrichment GO terms for both the cancer versus normal in the HUBCM and TCGA datasets. Top canonical pathways for differential hypermethylation and corresponding low RNA expression includes genes with biological function in hemidesmosome assembly, mammary gland development epidermis development, and hormone biosynthetic process. Similarly, top canonical pathways for differential hypomethylation and corresponding high RNA expression includes genes with biological function as negative regulators of macrophage differentiation, cAMP-dependent protein kinase inhibitor activity, protein destabilization and clathrin-coated vesicles membrane. Genomic landscape localization of these 5623 probes in both datasets as shown by a pie chart demonstrated differences in genomic localization in the HUBCM versus TCGA datasets. Higher frequency of promoter-related differential methylated CpG probes (71% of CpG probes) were observed in the TCGA dataset compared to 54% of HUBCM AA dataset located in the promoter region. Higher frequencies of promoter methylation suggests that these differential methylated CpGs may possibly play different roles in gene regulation as mentioned above. On the other hand, higher frequency of differential methylated CpG probes (30% of CpG probes) in the HUBCM dataset were located in intergenic region compared to only 18% found in the TCGA dataset. High levels of intergenic DNA methylation may alter chromatin structure and reduce the efficiency of polymerase II elongation[54], and rate of transcriptional elongation regulating alternative splicing[55]. In addition, intergenic DNA methylation can influence chromatin remodelling[56]; and methylation of gene bodies and can lead to alternative splicing and disease-causing mutations[45] suggesting varied potential DNA methylation effects and differences in PCa aetiology and progression in AA compared to EUR men. Hierarchical clustering analysis of CGI DNA methylation and RNA expression correlation in the cancer and normal prostate tissues used in the HUBCM and TCGA datasets demonstrated heterogeneity in methylation and RNA expression levels in both the normal and cancer samples. However, samples were clearly classified into different clusters based on methylation and RNA expression levels whereby high methylation level in PCa samples correlated with low RNA expression and low DNA methylation in normal prostate tumours was associated with high RNA expression in both the HUBCM and TCGA datasets. Similarly, PCa samples that showed hypomethylation correlated very well with high RNA expression in both the HUBCM and TCGA datasets. Thus, the analysis of the TCGA dataset provided confirmatory evidence for our findings. Gene ontology terms enrichment in both the HUBCM and TCGA datasets for differential CGI hypermethylation and concomitant low RNA expression were genes with biological functions in cell-cell adhesion, cell cycle arrest, negative regulator of transcription for RNA, calcium ion binding and wound healing pathways. On the other hand, GO enrichment for CGI hypomethylation and concomitant high RNA expression were genes with biological functions in pathways involved in ligase activity, gene silencing by RNA, protein targeting to ER, histidine biosynthetic process and methylenetetrahydrofolate dehydrogenase (NAD+) activity.

Differential methylation changes that were associated with PCa progression as determined by Gleason score/tumour grade revealed 1655 probes that correlated significantly with Gleason score in both the HUBCM and TCGA datasets. Of the 1655 probes, 42 that corresponded to 25 unique genes demonstrated significant differential methylation with concomitant inverse RNA expression to be associated with Gleason score and these were considered biologically relevant for PCa progression. While the differential methylation and RNA expression levels were similar between the HUBCM and TCGA datasets, we identified more frequent and significant methylation changes of 12 genes that were uniquely associated with Gleason score in the HUBCM AA cohort. Several of these genes either directly or their transcriptional activity falls in two predominant pathways: glucocorticoid receptor signalling and tumour microenvironment pathways indicating that these two pathways could be important players in prostate cancer progression in our HUBCM AA cohort.

Steroid hormone receptors (SRs) signals have a multitude of functions in human biology and disease progression. The SR family of related ligand-activated transcription factors includes androgen, oestrogen, glucocorticoid, mineralocorticoid, and progesterone receptors. Crosstalk between related SRs allows them to modulate signalling and transcriptional responses to noncognate ligands. This flexibility can lead to altered genomic binding and subsequent changes in SR target gene expression. Studies have shown that AR and glucocorticoid receptor (GR) share the same chromatin binding sites and GR can regulate genes in the AR pathway[57]. Activation of GR can drive transcription of AR-related genes and restore downstream signalling in PCa[58] and increase resistance to antiandrogens bypassing androgen receptor blockade. The composition of the tumour microenvironment (TME) is complicated. It includes not only cancer cells, extracellular matrix, fibroblasts, endothelial and adipocytes, but also immune cells such as T-cells, NK cells, regulatory T (Treg) cells, tumour-associated macrophages (TAMs), and dendritic cells (DCs). Within the TME, cytokines and chemokines, together with a dynamic immunosuppressive network formed by the interaction of immune cells and tumour cells, interrupt immunotherapies and promote cancer progression across all stages of tumorigenesis[59]. Our observation of epigenetic changes associated with tumour microenvironment in AA PCa with higher Gleason score suggests a relationship between differential methylation profiles to ultimately modulate tumour environment and increase prostate aggression in AA patients.

Strengths of the study includes the large sample size of PCa and normal prostate tissues from our HUBCM cohort which increase statistical power of analysis. Our epigenetic DNA methylation results in AA samples were largely validated by the existing TCGA dataset. Some of the limitations in our comparative analysis with the TCGA AA dataset is that there were only 3 PCa AA methylation profiles and 7 normal AA mRNA profiles so statistical power might be limited, and this did not all direct comparison of the HUBCM AA dataset with the TCGA AA dataset. On the other hand, the TCGA dataset has a lot higher Gleason grade EUR samples than the HUBCM cohort and could confound statistical interpretation. In addition, categorizing patients into AA and EUR may overlook admixture from multiple genetic ancestries that could also confound current results.

## Conclusion

We report for the first time a comprehensive analysis of genome-wide differential methylation between AA and EUR in prostate. Additional studies that profile large numbers of well-matched tumours from AA and non-AA men from the same clinical setting as well as validation of our findings using in vitro approaches with clinical specimen from prostate cancer patients belonging to different ethnic groups will be needed to confirm the novel associations observed in this study and to provide insight into the clinical relevance of DNA methylation and prostate cancer disparity. Ultimately, as cancer genomic studies expand to include molecular profiling datasets from large prostate tissues from different ethnic groups, this would allow us to examine additional features such as mutation, copy-number variation, rearrangements and gene fusion to fully understand the biological contribution to different outcomes in prostate cancer patients of different ethnic groups.

## Data Availability

Any data that support the finding of this study are available from the corresponding author, BKA upon reasonable non-commercial request.

## References

[1] DeSantis CE, Siegel RL, Sauer AG, et al. Cancer statistics for African Americans, 2016: progress and opportunities in reducing racial disparities. CA Cancer J Clin. 2016;66(4):290–14. 26910411 10.3322/caac.21340

[2] Ward E, Jemal A, Cokkinides V, et al. Cancer disparities by race/ethnicity and socioeconomic status. CA Cancer J Clin. 2004;54(2):78–93. 15061598 10.3322/canjclin.54.2.78

[3] Hoffman RM, Gilliland FD, Eley JW, et al. Racial and ethnic differences in advanced-stage prostate cancer: the prostate cancer outcomes study. J Natl Cancer Inst. 2001;93(5):388–395. 11238701 10.1093/jnci/93.5.388

[4] Mahal BA, Aizer AA, Ziehr DR, et al. Trends in disparate treatment of African American men with localized prostate cancer across National Comprehensive cancer network risk groups. Urology. 2014;84(2):386–392. 24975710 10.1016/j.urology.2014.05.009

[5] Mahal BA, Berman RA, Taplin ME, et al. Prostate cancer-specific mortality across gleason scores in black vs nonblack men. JAMA. 2018;320(23):2479–2481. 30561471 10.1001/jama.2018.11716

[6] Baca SC, Prandi D, Lawrence MS, et al. Punctuated evolution of prostate cancer genomes. Cell. 2013;153(3):666–677. 23622249 10.1016/j.cell.2013.03.021 PMC3690918

[7] Barbieri CE, Demichelis F, Rubin MA. Molecular genetics of prostate cancer: emerging appreciation of genetic complexity. Histopathology. 2012;60(1):187–198. 22212086 10.1111/j.1365-2559.2011.04041.x

[8] Berger MF, Lawrence MS, Demichelis F, et al. The genomic complexity of primary human prostate cancer. Nature. 2011;470(7333):214–220. 21307934 10.1038/nature09744 PMC3075885

[9] Taylor BS, Schultz N, Hieronymus H, et al. Integrative genomic profiling of human prostate cancer. Cancer Cell. 2010;18(1):11–22. [published Online First: 20100624]. 20579941 10.1016/j.ccr.2010.05.026 PMC3198787

[10] Hieronymus H, Schultz N, Gopalan A, et al. Copy number alteration burden predicts prostate cancer relapse. Proc Natl Acad Sci U S A. 2014;111(30):11139–11144. [published Online First: 20140714]. 25024180 10.1073/pnas.1411446111 PMC4121784

[11] Barbieri CE, Baca SC, Lawrence MS, et al. Exome sequencing identifies recurrent SPOP, FOXA1 and MED12 mutations in prostate cancer. Nat Genet. 2012;44(6):685–689. [published Online First: 20120520]. 22610119 10.1038/ng.2279 PMC3673022

[12] Tomlins SA, Bjartell A, Chinnaiyan AM, et al. ETS gene fusions in prostate cancer: from discovery to daily clinical practice. Eur Urol. 2009;56(2):275–286. [published Online First: 20090424]. 19409690 10.1016/j.eururo.2009.04.036

[13] Carter HB, Albertsen PC, Barry MJ, et al. Early detection of prostate cancer: AUA Guideline. J Urol. 2013;190(2):419–426. [published Online First: 20130506]. 23659877 10.1016/j.juro.2013.04.119 PMC4020420

[14] Khani F, Mosquera JM, Park K, et al. Evidence for molecular differences in prostate cancer between African American and Caucasian men. Clin Cancer Res. 2014;20(18):4925–4934. 25056375 10.1158/1078-0432.CCR-13-2265 PMC4167562

[15] Shivapurkar N, Gazdar AF. DNA methylation based biomarkers in non-invasive cancer screening. Curr Mol Med. 2010;10(2):123–132. 20196733 10.2174/156652410790963303 PMC3397200

[16] Bree KK, Hensley PJ, Pettaway CA. Germline Predisposition to prostate cancer in diverse populations. Urol Clin North Am. 2021;48(3):411–423. 34210495 10.1016/j.ucl.2021.03.008

[17] Borno ST, Fischer A, Kerick M, et al. Genome-wide DNA methylation events in TMPRSS2-ERG fusion-negative prostate cancers implicate an EZH2-dependent mechanism with miR-26a hypermethylation. Cancer Discov. 2012;2(11):1024–1035. 22930729 10.1158/2159-8290.CD-12-0041

[18] Mahapatra S, Klee EW, Young CY, et al. Global methylation profiling for risk prediction of prostate cancer. Clin Cancer Res. 2012;18(10):2882–2895. 22589488 10.1158/1078-0432.CCR-11-2090

[19] Herman JG, Baylin SB. Gene silencing in cancer in association with promoter hypermethylation. N Engl J Med. 2003;349(21):2042–2054. 14627790 10.1056/NEJMra023075

[20] Baylin SB, Jones PA. A decade of exploring the cancer epigenome - biological and translational implications. Nat Rev Cancer. 2011;11(10):726–734. [published Online First: 20110923]. 21941284 10.1038/nrc3130 PMC3307543

[21] Lin X, Tascilar M, Lee WH, et al. GSTP1 CpG island hypermethylation is responsible for the absence of GSTP1 expression in human prostate cancer cells. Am J Pathol. 2001;159(5):1815–1826. 11696442 10.1016/S0002-9440(10)63028-3 PMC1867052

[22] Woodson K, O’Reilly KJ, Hanson JC, et al. The usefulness of the detection of GSTP1 methylation in urine as a biomarker in the diagnosis of prostate cancer. J Urol. 2008;179(2):508–511. discussion 11-2. 18076912 10.1016/j.juro.2007.09.073

[23] Jeronimo C, Henrique R, Hoque MO, et al. A quantitative promoter methylation profile of prostate cancer. Clin Cancer Res. 2004;10(24):8472–8478. 15623627 10.1158/1078-0432.CCR-04-0894

[24] Locke WJ, Guanzon D, Ma C, et al. DNA methylation cancer biomarkers: translation to the clinic. Front Genet. 2019;10:1150. [published Online First: 20191114]. 31803237 10.3389/fgene.2019.01150 PMC6870840

[25] Massie CE, Mills IG, Lynch AG. The importance of DNA methylation in prostate cancer development. J Steroid Biochem Mol Biol. 2017;166:1–15. [published Online First: 20160424]. 27117390 10.1016/j.jsbmb.2016.04.009

[26] Lee WH, Morton RA, Epstein JI, et al. Cytidine methylation of regulatory sequences near the pi-class glutathione S-transferase gene accompanies human prostatic carcinogenesis. Proc Natl Acad Sci U S A. 1994;91(24):11733–11737. 7972132 10.1073/pnas.91.24.11733 PMC45306

[27] Mian OY, Khattab MH, Hedayati M, et al. GSTP1 Loss results in accumulation of oxidative DNA base damage and promotes prostate cancer cell survival following exposure to protracted oxidative stress. Prostate. 2016;76(2):199–206. [published Online First: 20151008]. 26447830 10.1002/pros.23111 PMC4734373

[28] Geybels MS, Zhao S, Wong CJ, et al. Epigenomic profiling of DNA methylation in paired prostate cancer versus adjacent benign tissue. Prostate. 2015;75(16):1941–1950. [published Online First: 20150918]. 26383847 10.1002/pros.23093 PMC4928710

[29] Kobayashi Y, Absher DM, Gulzar ZG, et al. DNA methylation profiling reveals novel biomarkers and important roles for DNA methyltransferases in prostate cancer. Genome Res. 2011;21(7):1017–1027. [published Online First: 20110426]. 21521786 10.1101/gr.119487.110 PMC3129245

[30] Partin AW, Van Neste L, Klein EA, et al. Clinical validation of an epigenetic assay to predict negative histopathological results in repeat prostate biopsies. J Urol. 2014;192(4):1081–1087. [published Online First: 20140418]. 24747657 10.1016/j.juro.2014.04.013 PMC4337855

[31] Partin AW, Criekinge WA, Trock BJ, et al. Clinical evaluation of an epigenetic assay to predict missed cancer in prostate biopsy specimens. Trans Am Clin Climatol Assoc. 2016;127:313–327. 28066067 PMC5216473

[32] Woodson K, Hayes R, Wideroff L, et al. Hypermethylation of GSTP1, CD44, and E-cadherin genes in prostate cancer among US blacks and whites. Prostate. 2003;55(3):199–205. 12692786 10.1002/pros.10236

[33] Woodson K, Hanson J, Tangrea J. A survey of gene-specific methylation in human prostate cancer among black and white men. Cancer Lett. 2004;205(2):181–188. 15036650 10.1016/j.canlet.2003.11.027

[34] Enokida H, Shiina H, Urakami S, et al. Ethnic group-related differences in CpG hypermethylation of the GSTP1 gene promoter among African-American, Caucasian and Asian patients with prostate cancer. Int J Cancer. 2005;116(2):174–181. 15800905 10.1002/ijc.21017

[35] Das PM, Ramachandran K, Vanwert J, et al. Methylation mediated silencing of TMS1/ASC gene in prostate cancer. Mol Cancer. 2006;5:28. [published Online First: 20060718]. 16848908 10.1186/1476-4598-5-28 PMC1543653

[36] Kwabi-Addo B, Wang S, Chung W, et al. Identification of differentially methylated genes in normal prostate tissues from African American and Caucasian men. Clin Cancer Res. 2010;16(14):3539–3547. [published Online First: 20100706]. 20606036 10.1158/1078-0432.CCR-09-3342

[37] Tang D, Kryvenko ON, Mitrache N, et al. Methylation of the RARB gene increases prostate cancer risk in black Americans. J Urol. 2013;190(1):317–324. [published Online First: 20130130]. 23376149 10.1016/j.juro.2013.01.083 PMC3779133

[38] Sharad S, Ravindranath L, Haffner MC, et al. Methylation of the PMEPA1 gene, a negative regulator of the androgen receptor in prostate cancer. Epigenetics. 2014;9(6):918–927. [published Online First: 20140402]. 24694733 10.4161/epi.28710 PMC4065188

[39] Devaney JM, Wang S, Furbert-Harris P, et al. Genome-wide differentially methylated genes in prostate cancer tissues from African-American and Caucasian men. Epigenetics. 2015;10(4):319–328. [published Online First: 20150411]. 25864488 10.1080/15592294.2015.1022019 PMC4622564

[40] Ittmann M, Wieczorek R, Heller P, et al. Alterations in the p53 and MDM-2 genes are infrequent in clinically localized, stage B prostate adenocarcinomas. Am J Pathol. 1994;145(2):287–293. 8053489 PMC1887392

[41] Zhang L, Wang J, Wang Y, et al. MNX1 is oncogenically upregulated in African-American prostate cancer. Cancer Res. 2016;76(21):6290–6298. 27578002 10.1158/0008-5472.CAN-16-0087 PMC5093064

[42] Creighton CJ, Nagaraja AK, Hanash SM, et al. A bioinformatics tool for linking gene expression profiling results with public databases of microRNA target predictions. RNA. 2008;14(11):2290–2296. [published Online First: 20080923]. 18812437 10.1261/rna.1188208 PMC2578856

[43] Saldanha AJ. Java Treeview–extensible visualization of microarray data. Bioinformatics. 2004;20(17):3246–3248. [published Online First: 20040604]. 15180930 10.1093/bioinformatics/bth349

[44] Cancer Genome Atlas Research Network. The molecular taxonomy of primary prostate cancer. Cell. 2015;163:1011–1025. 26544944 10.1016/j.cell.2015.10.025 PMC4695400

[45] Jones PA. Functions of DNA methylation: islands, start sites, gene bodies and beyond. Nat Rev Genet. 2012;13(7):484–492. [published Online First: 20120529]. 22641018 10.1038/nrg3230

[46] Baylin SB, Herman JG, Graff JR, et al. Alterations in DNA methylation: a fundamental aspect of neoplasia. Adv Cancer Res. 1998;72:141–196. 9338076

[47] Stopsack KH, Nandakumar S, Arora K, et al. Differences in prostate cancer genomes by self-reported race: contributions of genetic ancestry, modifiable cancer risk factors, and clinical factors. Clin Cancer Res. 2021. DOI:10.1158/1078-0432.CCR-21-2577. [published Online First: 20211019]. PMC8776579 34667026

[48] Martin DN, Starks AM, Ambs S. Biological determinants of health disparities in prostate cancer. Curr Opin Oncol. 2013;25(3):235–241. 23399519 10.1097/CCO.0b013e32835eb5d1 PMC6278601

[49] Lara OD, Wang Y, Asare A, et al. Pan-cancer clinical and molecular analysis of racial disparities. Cancer. 2020;126(4):800–807. [published Online First: 20191115]. 31730714 10.1002/cncr.32598 PMC6992510

[50] Wang BD, Ceniccola K, Yang Q, et al. Identification and functional validation of reciprocal microRNA-mRNA pairings in African American prostate cancer disparities. Clin Cancer Res. 2015;21(21):4970–4984. [published Online First: 20150618]. 26089375 10.1158/1078-0432.CCR-14-1566 PMC4631799

[51] Spratt DE, Chan T, Waldron L, et al. Racial/Ethnic Disparities in Genomic Sequencing. JAMA Oncol. 2016;2(8):1070–1074. 27366979 10.1001/jamaoncol.2016.1854 PMC5123755

[52] Fan X, Shao L, Fang H, et al. Cross-platform comparison of microarray-based multiple-class prediction. PLoS One. 2011;6(1):e16067. [published Online First: 20110111]. 21264309 10.1371/journal.pone.0016067 PMC3019174

[53] Yauk CL, Berndt ML, Williams A, et al. Comprehensive comparison of six microarray technologies. Nucleic Acids Res. 2004;32(15):e124. [published Online First: 20040827]. 15333675 10.1093/nar/gnh123 PMC516080

[54] Lorincz MC, Dickerson DR, Schmitt M, et al. Intragenic DNA methylation alters chromatin structure and elongation efficiency in mammalian cells. Nat Struct Mol Biol. 2004;11(11):1068–1075. [published Online First: 20041003]. 15467727 10.1038/nsmb840

[55] Kornblihtt AR. Chromatin, transcript elongation and alternative splicing. Nat Struct Mol Biol. 2006;13(1):5–7. 16395314 10.1038/nsmb0106-5

[56] Suzuki MM, Bird A. DNA methylation landscapes: provocative insights from epigenomics. Nat Rev Genet. 2008;9(6):465–476. 18463664 10.1038/nrg2341

[57] Sahu B, Laakso M, Pihlajamaa P, et al. FoxA1 specifies unique androgen and glucocorticoid receptor binding events in prostate cancer cells. Cancer Res. 2013;73(5):1570–1580. [published Online First: 20121226]. 23269278 10.1158/0008-5472.CAN-12-2350

[58] Arora VK, Schenkein E, Murali R, et al. Glucocorticoid receptor confers resistance to antiandrogens by bypassing androgen receptor blockade. Cell. 2013;155(6):1309–1322. 24315100 10.1016/j.cell.2013.11.012 PMC3932525

[59] Tang H, Qiao J, Fu YX. Immunotherapy and tumor microenvironment. Cancer Lett. 2016;370(1):85–90. [published Online First: 20151019]. 26477683 10.1016/j.canlet.2015.10.009 PMC4725050

